# A Case of Imperforate Hymen*Read before the Tri-State Medical Society of Alabama Georgia, and Tennessee.

**Published:** 1894-09

**Authors:** Andrew Boyd

**Affiliations:** Scottsboro, Ala.; Censor and Secretary of Jackson County Medical Society; Ex-Vice-President of the Tri-State Medical Society of Alabama, Georgia, and Tennessee; Member of Alabama Medical Association; Surgeon Third Regiment Alabama State Troops


### A CASE OF IMPERFORATE HYMEN.*

BY ANDREW BOYD., M.D., SCOTTSBORO, ALA.,

Censor and Secretary of Jackson County Medical Society; Ex-Vice-President of the Tri-State Medical Society of Alabama, Georgia, and Tennessee; Member of Alabama Medical Association; Surgeon Third Regiment Alabama State Troops.

This case of imperforate hymen, I think, will be quite interesting on account of its rarity. The case was first seen last November, nearly a year ago, by another physician. He was called to see some other member of the family and was asked to prescribe for this young lady, our patient.

* Read before the Tri-State Medical Society of Alabama Georgia, and Tennessee.

He found her to be about sixteen years old, the eldest of five healthy children, was going to school, robust, stout and to all appearances healthy, very quiet and modest in her manners. Her only complaint then was pain in the lower limbs and arms, some constipation, headache, with vertigo. Not a symptom then pointing, as afterwards found, to her true trouble. She was prescribed for and got along very well until the latter part of December, when all the above symptoms were exaggerated, the pain in the limbs, as she described it, was very great, excruciating, had several degrees of fever, was constipated. The myalgia, however, was her great trouble, and this was felt in the upper and lower limbs only, but more in the lower than the upper. No symptoms yet complained of that pointed to the true trouble. Her mother was asked, however, about her menstrual flow during this visit. She said the girl had never seen anything, but her sister, eighteen months younger, had been menstruating regularly for the last four months, had begun when a little over fourteen years old. The mother was questioned closely concerning the symptoms of suppressed or retained menstruation. They were *nil*, except the pain in the lower extremities. Quinine was prescribed for the fever and the compound aloin pill, one three times a day, was given for the constipation. She was in school all the time, except every two, three, four and sometimes five weeks she would become ill with the symptoms already named, be in bed a day or two and then up, but the myalgia was at all times more or less present, and she had all the symptoms of anemia except her rosy complexion and robust form.

This went on until some time in May, when her mother discovered that her abdomen was much larger than it had been, in fact much too large for a schoolgirl. This enlargement certainly had been going on for some months, but no symptom had ever indicated that the trouble was in the pelvis. She was seen by two physicians, and a thorough examination made except *per vaginam*, I will state that at this time all the symptoms complained of before were much increased. Her temperature for several days ran up as high as 103°, circulation and respiration in proportion. Her symptoms resembled a remittent fever very much. She was so treated and the abdominal enlargement looked upon with suspicion. She had in the past few weeks got up in the morning sick at the stomach, and the mammæ were thought to be a little larger than formally. Very few questions were asked and not a very searching examination made of this abdominal enlargement, for it did look suspicious. It was thought wise to treat the present symptoms and this would take care of itself. The pelvis looked as if it contained a fetus about four months old. I will say that at this time, June the 4th, there was the first intimation that there was any trouble with the pelvic organs; the myalgia and constipation had been all the symptoms complained of, and this myalgia not even in the muscles of the back.

This fever lasted for four or five days. She convalesced slowly and did not come out of this attack as well and as rapidly as she had former ones. She now began to complain of great pain in the back and the pain was greatly increased in the limbs. Constipation was stubborn and some pain and trouble on micturition. The poor girl went on from this time, June the 4th, perhaps not all the time, but certainly a great part of it, suffering excruciating pain we know, until August the 15th. From June the 4th to the time of operation, August 15th, she had several of these febrile attacks as described. She was treated mostly by her mother. No great stress as yet had been placed on her never having menstruated, but now it began to assume a serious aspect. Here a stout, sixteen-year-old girl, with healthy parents, a sister, nearly two years her junior, menstruating regularly. There must be some cause for this, and, furthermore, there must be some cause for these febrile attacks coming on every two, three or four weeks with such excruciating pain. And then, why this enlarged abdomen?

So on August the 15th, a thorough examination was demanded, and after much persuasion, was granted. She was anesthetized, and a tough perfect hymen was found protruding from the vagina. This was incised with a bistuory. The opening was made about one and one-half inches in length, and between five and six quarts of dark, tarry menstrual fluid escaped, no clots, but all of it was very thick, except perhaps about a pint, which was the last to come away. The fluid was fully a half an hour coming away. A large cavity was seen just inside of the vagina, the uterus and everything was pushed upwards, leaving the pelvic walls perfectly bare, and exposing a cavity large enough in which to place the largest size cocoanut. The uterus was pushed away up even with the umbilicus, the os was widely dilated and blood oozing freely from it. I think the greater part of this fluid that came away last came from the uterus.

We allowed the entire amount to flow away, and the parts were washed out with a bichloride solution, 1 in 5000. We used a fountain syringe, and threw the solution in until it came back clear. A drainage tube was then placed therein, and packed with iodoform gauze. The parts were washed out every day with the bichloride solution.

She got along finely without a single bad symptom until the ninth day, when she had a rigor, and the temperature ran up to 105°; abdomen very tender and tympanitic, with all the symptoms of local peritonitis. The saline treatment, as ordered and used by our worthy President, as the only thing that offered relief, except the knife, was given as directed by him to its fullest extent, together with just enough morphia to quiet the pain. The fever was soon reduced, the inflammation of the bowels quieted, and she made an easy and a rapid recovery, and is now, over two months since the operation, a stout, healthy, robust girl going to school, and better than all, menstruating regularly without any pain or discomfort whatever.

---

COLD DRESSINGS.—Dr. Gregory Doyle (*Buffalo Medical and Surgical Journal*) says that the common practice of applying cold water dressings to injured or fractured limbs cannot be too strongly deprecated, although sanctioned by many surgical authors. Cold applications may, while in actual contact, keep down the temperature, but on their removal an injurious reaction is very apt to occur. A surgical experience of nearly thirty years has taught him that warm applications only should be used in the treatment of local inflammations.

---

THE HEPATIC STIMULANT.—Dr. R. R. Grimes, Severy, Kan., says: Chionia is *the* remedy when a hepatic stimulant is indicated. want no other, for there is none better.—*Ex.*

